# Removal of a giant intrathoracic cyst from the anterior mediastinum

**DOI:** 10.1186/s13019-014-0152-2

**Published:** 2014-09-20

**Authors:** Wobbe Bouma, Theo J Klinkenberg, Caroline Van De Wauwer, Wim Timens, Massimo A Mariani

**Affiliations:** Department of Cardiothoracic Surgery, University of Groningen, University Medical Center Groningen, Groningen, The Netherlands; Department of Pathology, University of Groningen, University Medical Center Groningen, Groningen, The Netherlands

**Keywords:** Intrathoracic mesothelial cyst, Mediastinal tumor, Surgical removal

## Abstract

A 45-year-old caucasian man with progressive dyspnea appeared to have a giant intrathoracic cyst in the anterior mediastinum encasing the heart and compressing both lungs. He underwent succesful removal of the cyst through a median sternotomy. Recovery was uneventful. Gross examination revealed a thin-walled cyst filled with clear fluid. Microscopic histopathologic examination revealed a cyst wall lined by cubic cells and underlying loose connective tissue with remnants of thymic tissue. The definitive diagnosis was an intrathoracic (simple) mesothelial cyst. An intrathoracic mesothelial cyst is a benign, generally asymptomatic tumor that can be located in the anterior cardiophrenic angle, the paravertebral or paratracheal regions, or in the anterior mediastinum. It can become rather large before it becomes symptomatic, at which point surgical removal is generally warranted.

**Electronic supplementary material:**

The online version of this article (doi:10.1186/s13019-014-0152-2) contains supplementary material, which is available to authorized users.

## Background

An intrathoracic mesothelial cyst is a congenital abnormality and represents 3-6% of mediastinal tumors [1],[2]. These cysts are generally asymptomatic [3] and located in the anterior cardiophrenic angle [2],[4], but can also be found in the paravertebral or paratracheal regions [2] or in the anterior mediastinum, as shown in this case.

## Case presentation

**Figure 1:**
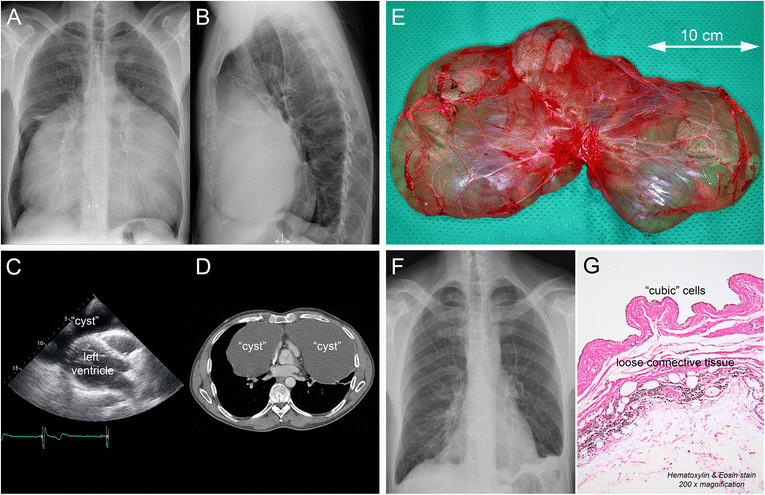
**Imaging.** Preoperative posterioranterior **(A)** and lateral **(B)** chest X-ray showing a large mass in the anterior mediastinum with posterior displacement of the heart. Transthoracic echocardiography **(C)** showing a large echolucent space around the heart (parasternal long-axis view). Computertomography **(D)** showing a large cyst in the anterior mediastinum encasing the heart with compression of both lungs (transverse view at the level of the aortic valve). Gross examination **(E)** revealed a thin-walled cyst filled with clear fluid. A postoperative posterioranterior chest X-ray **(F)** showed a remarkable improvement and a normal cardiac silhouette. Microscopic histopathologic examination **(G)** showed a cyst wall lined by cubic cells and underlying loose connective tissue with remnants of thymic tissue.

A 45-year-old caucasian man with no significant past medical history was referred to our institution with progressive dyspnea. A chest X-ray showed a large mass in the anterior mediastinum (Figure 1A,B). Transthoracic echocardiography showed a large echolucent space around the heart (Figure 1C; parasternal long-axis view). Computertomography showed a large cyst in the anterior mediastinum encasing the heart (Figure 1D; transverse view at the level of the aortic valve). The cyst was 33 × 22 × 4 cm in size with a radiodensity of 12 to 18 Hounsfield units.

The patient chose to have the cyst removed due to its symptomatic nature and he underwent successful removal of the giant cyst through a median sternotomy. Due to the location and size of the cyst and in order to keep the cyst intact during resection, we were required to perform a median sternotomy. The cyst did not involve the pericardium or other surrounding structures. Only mild adhesions were encountered and the cyst was left intact during resection. The cyst was thin-walled and filled with clear fluid (Figure 1E). A postoperative chest X-ray showed a remarkable improvement and a normal cardiac silhouette (Figure 1F). Postoperative recovery was uneventful with resolution of the patient’s symptoms.

Microscopic histopathologic examination (Figure 1G) showed a benign cyst (wall) lined by cubic cells and underlying loose connective tissue with remnants of thymic tissue. Although remnants of thymic tissue were found, the cyst did not seem to originate from the thymus, as thymic cysts are generally lined by (simple) squamous epithelium. The definitive diagnosis was an intrathoracic (simple) mesothelial cyst.

## Discussion

An intrathoracic mesothelial (or “coelomic”) cyst is a congenital abnormality. Little is known about the exact embryology, but mesothelial cysts are hypothesized to occur as a result of an anomaly in the development of the pericardial coelom [5]. Mesothelial cysts comprise 3-6% of mediastinal tumors and are usually diagnosed when patients are between 40 and 60 years of age [1],[2]. An intrathoracic mesothelial cyst is a benign tumor that can become rather large before it becomes symptomatic.

The optimal management of intrathoracic mesothelial cysts is unknown. Due to its benign nature most asymptomatic cysts only require serial follow-up imaging [6]. Symptomatic cysts usually require treatment. Percutaneous ultrasound-guided or computed tomography-guided needle drainage of the cyst can be performed when symptoms are mild or if cytologic evaluation is required [4],[7]. Cysts with more severe symptoms or complex cysts (irregular, multi-loculated, unusual location) require surgical resection [4]. Surgical resection can usually be performed with a limited thoracotomy or a videothoracoscopic procedure [2],[8], but sometimes a median sternotomy may be required.

## Conclusions

An intrathoracic mesothelial cyst is a benign, generally asymptomatic tumor that can be located in the anterior cardiophrenic angle, the paravertebral or paratracheal regions, or in the anterior mediastinum. It can become rather large before it becomes symptomatic, at which point surgical removal is generally warranted.

## Consent

Written informed consent was obtained from the patient for publication of this case report and any accompanying images. A copy of the written consent is available for review by the Editor-in-Chief of this journal.

## Authors’ original submitted files for images

Below are the links to the authors’ original submitted files for images. Authors’ original file for figure 1
