# Supplementary figures and images for: Removal of a giant intrathoracic cyst from the anterior mediastinum
Source: J Cardiothorac Surg. 2014 Sep 20;9:152. doi: 10.1186/s13019-014-0152-2 (PMC4182827; doi:10.1186/s13019-014-0152-2)

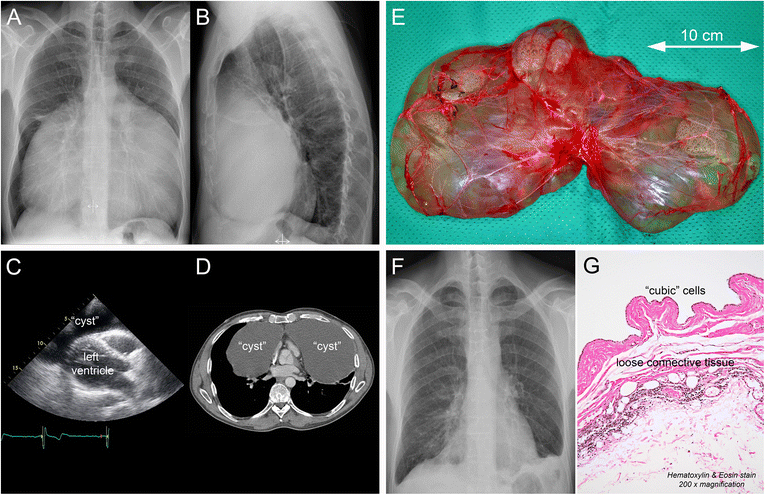

Supplement: Supplementary file 1 — Authors’ original file for figure 1 [file 13019_2014_152_MOESM1_ESM.gif]
